# Protective Effects of Dietary Epigallocatechin-3-Gallate in Weaned Pigs Exposed to Oxidative Stress

**DOI:** 10.3390/antiox15101270

**Published:** 2026-10-02

**Authors:** Qiming Duan, Xiang Li, Kunhong Xie, Aibing Yu, Yuheng Luo, Ping Zheng, Xiangbing Mao, Hui Yan, Jun He

**Affiliations:** 1 Institute of Animal Nutrition, Sichuan Agricultural University, Chengdu 611130, China; 2022114016@stu.sicau.edu.cn (Q.D.); xiangli@sicau.edu.cn (X.L.); xiekunhong@sicau.edu.cn (K.X.); yhluo@sicau.edu.cn (Y.L.); pingzheng@sicau.edu.cn (P.Z.); 3856@sicau.edu.cn (X.M.); yan.hui@sicau.edu.cn (H.Y.); 2 Key Laboratory of Animal Nutrition and Efficient Feed Utilization, Chengdu 611130, China; 3 Newhope Co., Ltd., Chengdu 610011, China

**Keywords:** Diquat, intestinal barrier, mucosal immunity, T-cell phenotype, antioxidant capacity

## Abstract

Epigallocatechin-3-gallate (EGCG), a major catechin in green tea, possesses antioxidant and immunomodulatory properties. We hypothesized that dietary EGCG supplementation before oxidative challenge would attenuate Diquat-induced redox disturbance and help maintain intestinal epithelial integrity and immune-related homeostasis in weaned pigs. To test this hypothesis, 32 weaned piglets were assigned to a 2 × 2 factorial arrangements involving dietary EGCG supplementation (0 or 50 mg/kg) and saline or Diquat challenge (10 mg/kg body weight). Dietary EGCG increased serum superoxide dismutase activity and immunoglobulin A, G, and M concentrations and decreased serum IL-1β, whereas Diquat decreased superoxide dismutase activity and immunoglobulin concentrations and increased TNF-α (*p* < 0.05). A significant EGCG × Diquat interaction was detected for serum total antioxidant capacity (*p* = 0.020), which was increased by EGCG in Diquat-challenged pigs (Sidak-adjusted *p* = 0.001). Significant interactions were also observed for duodenal and jejunal ZO-1 immunoreactivity, jejunal sIgA immunoreactivity, and jejunal epithelial apoptosis (*p* < 0.05). Under Diquat challenge, EGCG increased ZO-1 and sIgA immunoreactivity and reduced TUNEL-positive staining (adjusted *p* < 0.05). Intestinal gene-expression responses were segment dependent: EGCG was associated with increased *IL-10* and *FOXP3* expression and with reduced expression of *IL-17*, *IL-22*, *RORγT*, *STAT3*, *caspase-3*, *caspase-9*, and *BAX* in several intestinal segments, particularly in Diquat-challenged pigs. Collectively, the observed findings indicate associations between dietary EGCG supplementation and systemic antioxidant and immune indices, epithelial barrier-related characteristics, immune-cell phenotypes, and mucosal transcriptional responses. Given the number of outcomes examined and the absence of a global multiplicity adjustment, these findings should be interpreted as exploratory and do not establish a specific protective mechanism.

## 1. Introduction

Weaning is a critical period in swine production during which piglets experience abrupt nutritional, environmental, and psychological changes [1,2,3]. These stressors can impair intestinal development and barrier function, reduce nutrient utilization, increase the incidence of diarrhea, and compromise growth performance [4,5,6,7,8,9]. Oxidative stress is considered an important contributor to weaning-associated intestinal dysfunction [10,11]. The transition from sow milk to plant-based solid diets, together with exposure to dietary and environmental stressors, may further increase oxidative pressure in the immature intestine [12,13,14]. Excessive production of reactive oxygen species (ROS) can damage cellular lipids, proteins, and DNA, disrupt tight-junction-associated epithelial integrity, and promote epithelial cell death [15,16,17,18,19]. Oxidative stress is also closely associated with impaired mucosal immune defense, including altered secretory immunoglobulin A (sIgA) responses and disturbances in T-cell-associated immune homeostasis [20,21]. Thus, oxidative injury to the intestinal epithelium involves not only disruption of the physical barrier but also changes in mucosal immune regulation.

Epigallocatechin-3-gallate (EGCG) is the predominant bioactive catechin in green tea (*Camellia sinensis*) and has attracted considerable interest because of its antioxidant and immunomodulatory properties [22,23]. Its multiple phenolic hydroxyl groups and galloyl moiety contribute to its free-radical-scavenging capacity [24]. Previous studies have shown that EGCG can influence oxidative and inflammatory responses by reducing ROS accumulation, supporting endogenous antioxidant defenses, and modulating inflammation-related signaling [25,26,27,28]. EGCG has also been associated with improved intestinal epithelial barrier function, including changes in tight-junction proteins and mucosal defense [29,30], and with modulation of CD4^+^ T-cell-associated responses [31,32]. These observations provide a biological basis for investigating EGCG as a dietary strategy to protect the intestinal epithelium during oxidative stress. However, in weaned pigs, it remains unclear whether dietary EGCG supplementation is concurrently associated with changes in systemic antioxidant status, intestinal epithelial integrity and apoptotic-related responses, and immune-cell phenotypes under oxidative stress conditions.

Diquat is a redox-cycling compound widely used to establish oxidative-stress models in pigs because it rapidly generates ROS and reproducibly induces systemic oxidative stress and intestinal epithelial injury [14]. This model therefore provides a controlled approach for evaluating the responses associated with dietary antioxidant intervention. We hypothesized that dietary EGCG supplementation would attenuate Diquat-associated oxidative injury in weaned pigs and would be accompanied by improved intestinal epithelial integrity and more favorable immune-related responses. Accordingly, the present study used a 2 × 2 factorial design to evaluate the effects of dietary EGCG supplementation and Diquat challenge on systemic antioxidant and immune indices, intestinal zonula occludens-1 and mucosal sIgA immunoreactivity, intestinal epithelial TUNEL-positive staining, T-cell phenotypes in the mesenteric lymph nodes and spleen, and intestinal immune- and apoptosis-related transcriptional profiles. The study was designed to characterize the epithelial and immunological responses associated with dietary EGCG supplementation under experimentally induced oxidative stress.

## 2. Materials and Methods

### 2.1. Animals, Diet, and Experimental Design

All experimental procedures were approved by the Institutional Animal Care and Use Committee of Sichuan Agricultural University (approval No. 20231043; approved on 15 September 2023). A total of 32 male Duroc × Landrace × Yorkshire piglets, weaned at 21 days of age and with an average initial body weight of 6.55 ± 0.22 kg, were used in a 2 × 2 factorial experiment involving dietary EGCG supplementation and Diquat challenge. The animal trial and sample collection were conducted in 2024 at Sichuan Agricultural University, China, and laboratory analyses were completed during 2024–2025. Before treatment allocation, the piglets were ranked according to initial body weight and arranged into eight blocks of four pigs with similar body weights. Within each block, the four pigs were randomly allocated to one of the four treatments using a computer-generated random-number sequence generated using the RAND function in Microsoft Excel 2019 (Microsoft Corporation, Redmond, WA, USA).This procedure resulted in eight pigs per treatment and maintained a similar initial-body-weight distribution among treatments. The four treatments were as follows: CON, basal diet without Diquat challenge; EGCG, basal diet supplemented with 50 mg/kg EGCG without Diquat challenge; DCON, basal diet followed by Diquat challenge; and DEGCG, basal diet supplemented with 50 mg/kg EGCG followed by Diquat challenge. The individual pig was considered the experimental unit. Technical replicates, repeated microscopic fields, and repeated measurements within the same biological sample were not treated as independent experimental units.

Blinding was not feasible for personnel responsible for diet preparation, animal management, and administration of Diquat or saline because these procedures required knowledge of treatment allocation. Before laboratory measurements, samples were relabeled using coded identifiers that did not reveal treatment identity. Investigators performing the laboratory assays and the acquisition and quantification of histological, immunohistochemical, immunofluorescence, and TUNEL images were unaware of treatment allocation until the corresponding measurements had been completed.

Prior to the experiment, all piglets received the same diet and routine management. During the experimental period, each piglet was housed individually in a stainless-steel cage (1.5 × 0.7 m), with one pig per cage. Each cage was equipped with an individual feeder and nipple drinker, and pigs had ad libitum access to feed and fresh water throughout the experiment. Room temperature was maintained at 25–28 °C, with a relative humidity of approximately 65% ± 5%. Routine husbandry, including cleaning and ventilation, was performed daily. Animals were observed daily during routine husbandry and health monitoring for general health and obvious signs of distress. No mortality occurred during the experimental period. A formal quantitative clinical adverse-event scoring system was not prospectively implemented.

Animals were observed at least once daily for general health, feed and water intake, mobility, responsiveness, respiratory difficulty, persistent recumbency, and other signs of pain or severe distress. Veterinary assessment and early euthanasia were to be undertaken if an animal exhibited severe and unrelieved pain or distress, inability to stand or obtain feed and water, persistent recumbency, severe respiratory distress, or a moribund condition. No animal met these intervention criteria, and no mortality or unscheduled euthanasia occurred during the experiment. A formal quantitative clinical adverse-event scoring system was not prospectively implemented.

All 32 pigs completed the experiment and were retained in the animal-level analyses. Animal-level exclusion criteria included death or euthanasia before scheduled sampling, a major illness or injury unrelated to the experimental treatments that prevented completion of the experiment, or failure to obtain an analyzable biological sample. Measurements for a particular endpoint were considered unavailable only when insufficient sample material or a technical assay failure prevented analysis. No animals were excluded at the animal level, and missing technical measurements, where present, were not imputed. The actual number of animals analyzed for each endpoint is reported in the corresponding table, figure, or Supplementary Statistical Table.

The dietary EGCG supplementation level of 50 mg/kg was selected based on preliminary dose–response experiments conducted in our laboratory, in which dietary EGCG was evaluated at 0, 25, 50, and 100 mg/kg, and the 50 mg/kg supplementation level showed the most favorable overall responses in growth performance and serum antioxidant status among the tested levels. The EGCG product was supplied by Guangdong Laiyin Biotechnology Co., Ltd. (Guangzhou, China), with a specified purity of ≥95%. A retained sample from the same batch used in the animal experiment was subsequently analyzed by high-performance liquid chromatography (HPLC) for compositional verification. Using external-standard quantification with diode-array detection at 280 nm, the EGCG content was determined to be 95.17% (Supplementary File S2).

Diquat dibromide monohydrate (analytical standard; catalog no. D101258) was purchased from Shanghai Aladdin Biochemical Technology Co., Ltd. (Shanghai, China). The Diquat challenge protocol was established with reference to a previous study conducted in our laboratory by Chen et al. [33]. Diquat dibromide monohydrate was dissolved in sterile normal saline to a final concentration of 10 mg/mL. On experimental day 15, after 14 days of dietary treatment, pigs in the Diquat-challenged groups received a single intraperitoneal injection of Diquat at 10 mg/kg body weight, corresponding to an injection volume of 1 mL/kg body weight, whereas pigs in the non-challenged groups received an equal volume of sterile normal saline by intraperitoneal injection. The challenge dose, route of administration, and timing were selected with reference to the Diquat-induced oxidative-stress model previously established in weaned pigs in our laboratory by Chen et al. [33].

Following the single Diquat challenge, pigs were maintained for a 7-day post-challenge observation period before sample collection on experimental day 22. Thus, the experimental schedule comprised a 14-day period of dietary EGCG supplementation before Diquat challenge followed by a 7-day post-challenge observation period, allowing evaluation of the responses associated with prior dietary EGCG supplementation under Diquat-induced oxidative stress.

The animals and experimental design used in the present study were the same as those previously described by Duan et al. [34], who reported nutritional and intestinal functional outcomes, including growth performance, nutrient digestibility, serum biochemical parameters, intestinal morphology and antioxidant status, intestinal microbial populations and metabolites, and the expression of selected genes related to epithelial barrier function, redox regulation, and inflammation. Because the growth-performance outcomes from this cohort were included in that publication, they are not reproduced or reanalyzed in the present manuscript. In contrast, the present study addresses a distinct scientific question and reports additional datasets focusing on systemic antioxidant and immune-related responses, intestinal ZO-1 immunoreactivity, mucosal sIgA immunoreactivity, TUNEL staining, flow-cytometric immune-cell phenotypes in the mesenteric lymph nodes and spleen, and intestinal immune- and apoptosis-related transcriptional responses. These outcomes were analyzed to further characterize the epithelial and immune responses associated with dietary EGCG supplementation under Diquat-induced oxidative stress.

The basal diet was prepared in accordance with the nutrient recommendations for swine provided by the NRC (2012) [35], and its composition is shown in Table 1.

### 2.2. Sample Collection

On day 22, blood samples were collected from all pigs (*n* = 8 per treatment) by jugular vein puncture. Blood intended for serum analyses was allowed to clot and then centrifuged at 3500× *g* for 10 min at 4 °C. The resulting serum was aliquoted and stored at −20 °C until analysis. Following blood collection, each pig was euthanized by an intravenous overdose of sodium pentobarbital at 100 mg/kg body weight. Death was confirmed by the absence of spontaneous respiration and heartbeat before tissue collection commenced. All intestinal and lymphoid tissues were collected only after death had been confirmed.

Segments of the duodenum, jejunum, and middle ileum were collected immediately after confirmation of death. Approximately 4-cm segments from the middle portion of each intestinal region were gently rinsed with cold phosphate-buffered saline and fixed in 4% paraformaldehyde for morphological and imaging analyses. Intestinal mucosa was scraped from the remaining intestinal segments using a sterile scalpel blade, transferred into cryogenic tubes, immediately frozen in liquid nitrogen, and stored at −80 °C until analysis. Mesenteric lymph nodes and spleen were collected immediately after death had been confirmed and processed fresh in ice-cold PBS for preparation of single-cell suspensions for flow-cytometric analysis.

### 2.3. Serum Antioxidant, Immunoglobulin, and Cytokine Indices

Serum biochemical measurements were performed using samples from eight pigs per treatment (*n* = 8), with the individual pig serving as the biological and experimental unit. Serum samples were analysed without predilution. Each biological sample was measured in triplicate, and the mean of the three technical replicates was used for statistical analysis.

Serum total antioxidant capacity (T-AOC), catalase (CAT), malondialdehyde (MDA), total superoxide dismutase (T-SOD), and glutathione peroxidase (GSH-Px) activity were determined using commercial assay kits from Nanjing Jiancheng Bioengineering Institute (Nanjing, China). The assays comprised T-AOC (colorimetric method; catalogue no. A015-1-2), CAT (visible-light method; catalogue no. A007-1-1), MDA (thiobarbituric acid method; catalogue no. A003-1-2), T-SOD (hydroxylamine method; catalogue no. A001-1-2), and GSH-Px (colorimetric method; catalogue no. A005-1-2). All measurements were performed according to the manufacturer’s instructions. In the MDA assay, MDA reacted with thiobarbituric acid to form a coloured product, and the absorbance was measured at 532 nm. The retained kit documentation did not specify fixed analytical ranges applicable to serum samples for T-AOC, CAT, MDA, T-SOD, or GSH-Px. T-AOC, CAT, T-SOD, and GSH-Px activities were expressed as U/mL, whereas MDA concentration was expressed as nmol/mL.

Serum immunoglobulin concentrations were measured using porcine-specific ELISA kits supplied by Jiangsu Meimian Industrial Co., Ltd. (Yancheng, China). The kits and manufacturer-stated analytical ranges were as follows: IgA, catalogue no. MM-0905O1, 0–36 μg/mL; IgG, catalogue no. MM-0403O1, 0–400 μg/mL; and IgM, catalogue no. MM-0402O1, 0–40 μg/mL. Serum inflammatory cytokine concentrations were measured using porcine-specific ELISA kits supplied by Quanzhou Rui Xin Biological Technology Co., Ltd. (Quanzhou, China), including IL-1β (catalogue no. RX501070P; analytical range, 25–640 pg/mL), IL-6 (catalogue no. RX501064P; 25–800 pg/mL), IL-17 (catalogue no. RX501074P; 1.5–48 pg/mL), and TNF-α (catalogue no. RX500888P; 6.25–200 pg/mL). All ELISA measurements were performed in triplicate according to the respective manufacturers’ protocols, and concentrations were calculated from the corresponding standard curves. Results were expressed as μg/mL for IgA, mg/mL for IgG and IgM, and pg/mL for IL-1β, IL-6, IL-17, and TNF-α.

### 2.4. Immunofluorescence Analysis of ZO-1

The localization and abundance of zonula occludens-1 (ZO-1) in the intestinal epithelium were determined by immunofluorescence staining. Paraformaldehyde-fixed intestinal samples were embedded in paraffin and cut into 4-μm-thick sections. For each pig, two non-consecutive sections separated by approximately 80–100 μm were analyzed. After deparaffinization and rehydration, antigen retrieval was performed using EDTA buffer (pH 9.0). The sections were blocked with 3% bovine serum albumin and incubated overnight at 4 °C with a rabbit anti-ZO-1 primary antibody (Servicebio, GB15195-100; 1:1000). After washing with PBS, sections were incubated with Cy3-conjugated goat anti-rabbit IgG (H + L) secondary antibody Servicebio (Wuhan, China; catalog no. GB21303; dilution 1:300) for 1 h at room temperature in the dark. Nuclei were counterstained with 4′,6-diamidino-2-phenylindole (DAPI).

For each section, five randomly selected, non-overlapping fields with intact and well-oriented mucosal structures were captured at 200× magnification using a confocal fluorescence microscope. All images within an intestinal segment were acquired under identical exposure and acquisition settings. The mean optical density of ZO-1 fluorescence within the intestinal epithelial region was quantified using ImageJ software (version 1.54h; National Institutes of Health, Bethesda, MD, USA), with the same background-subtraction and threshold settings applied to all images. The values obtained from the 10 fields were averaged to generate one value for each pig. Eight pigs were analyzed per treatment (*n* = 8), and the individual pig was considered the experimental unit.

### 2.5. Immunohistochemical Analysis of Mucosal sIgA

The abundance of sIgA in the intestinal mucosa was determined by immunohistochemical staining. Paraformaldehyde-fixed intestinal samples were embedded in paraffin and cut into 4-μm-thick sections. Two non-consecutive sections separated by approximately 80–100 μm were analyzed for each pig. After deparaffinization and rehydration, endogenous peroxidase activity was blocked with 3% H_2_O_2_ in methanol. Antigen retrieval was performed using citrate buffer (pH 6.0), followed by blocking with 10% goat serum. The sections were incubated overnight at 4 °C with an anti-pig sIgA primary antibody (Beijing Biosynthesis Biotechnology Co., Ltd., Beijing, China), followed by incubation with a biotinylated secondary antibody. Immunoreactive signals were developed using 3,3′-diaminobenzidine (DAB), with the color reaction monitored microscopically and terminated by washing, followed by hematoxylin counterstaining of nuclei.

For each section, five randomly selected, non-overlapping fields with intact and well-oriented mucosal structures were captured at 200× magnification using identical microscope settings. The integrated optical density and positive staining area of sIgA immunoreactivity within the intestinal mucosal region were quantified using Image-Pro Plus 6.0. Mean optical density was calculated as the integrated optical density divided by the positive staining area. The same image-analysis criteria were applied to all samples. The values obtained from the 10 fields were averaged to generate one value for each pig. Eight pigs were analyzed per treatment (*n* = 8), and the individual pig constituted the experimental unit.

### 2.6. TUNEL Assay

Intestinal epithelial TUNEL staining was performed using a terminal deoxynucleotidyl transferase-mediated dUTP nick-end labeling (TUNEL) assay kit (Servicebio, Wuhan, China; catalog no. G1504) according to the manufacturer’s instructions. Paraffin-embedded intestinal tissues were cut into 4-μm-thick sections, and two non-consecutive sections separated by approximately 80–100 μm were analyzed for each pig. The sections were deparaffinized, rehydrated, and subjected to the appropriate permeabilization treatment. The TUNEL reaction mixture was then applied, and the sections were incubated at 37 °C for 60 min in a humidified chamber protected from light. After washing with PBS, nuclei were counterstained with DAPI.

For each section, five randomly selected, non-overlapping fields containing intact and well-oriented intestinal epithelium were captured at 200× magnification using a fluorescence microscope. All images were acquired under identical microscope settings. The intestinal epithelial region was defined as the region of interest, and the mean optical density of TUNEL-positive signals was quantified using ImageJ software. The same background-subtraction and threshold settings were applied to all images. The values obtained from the 10 microscopic fields from the two sections were averaged to generate one biological value for each pig. Eight pigs were analyzed per treatment, and the individual pig was considered the experimental unit.

Because TUNEL-positive nuclei were not quantified relative to the total number of DAPI-positive nuclei, the TUNEL measurement was interpreted as TUNEL-positive signal intensity rather than the percentage of apoptotic cells.

### 2.7. Flow Cytometric Analysis of T-Cell Subsets in MLN and Spleen

Flow-cytometric analyses were performed using mesenteric lymph-node (MLN) and spleen samples from eight pigs per treatment. Fresh tissues were gently minced in ice-cold phosphate-buffered saline (PBS) and passed through sterile 70-μm cell strainers to obtain single-cell suspensions. After centrifugation, erythrocytes were lysed when necessary using an ammonium chloride-based erythrocyte lysis buffer. The remaining cells were washed and resuspended in staining buffer.

Single-cell suspensions were analyzed using a BD FACSVerse flow cytometer (BD Biosciences, San Jose, CA, USA). Separate antibody panels were used for CD3/CD4/CD8 surface phenotyping and intracellular FOXP3 or IL-17A detection.

For the CD3/CD4/CD8 surface-staining panel, lymphocytes were initially identified according to forward-scatter area (FSC-A) and side-scatter area (SSC-A), followed by singlet discrimination using FSC-A versus forward-scatter height (FSC-H). CD3^+^ cells were then gated, and CD4^+^ and CD8^+^ cell populations were quantified within the gated CD3^+^ population. Cells were stained with fluorochrome-conjugated antibodies against CD3, CD4, and CD8 (BD Pharmingen, San Diego, CA, USA; catalog nos. 559582, 559588, and 561475, respectively).

For intracellular FOXP3 and IL-17A analysis, separate aliquots of the cell suspensions were used. Lymphocytes were first identified according to FSC-A and SSC-A, followed by singlet discrimination using FSC-A versus FSC-H. CD4^+^ cells were subsequently gated, and FOXP3^+^ or IL-17A^+^ events were quantified separately within the gated CD4^+^ population after fixation and permeabilization. The antibodies used were anti-FOXP3 (clone FJK-16s; catalog no. 17-5773-82; Thermo Fisher Scientific, Waltham, MA, USA) and anti-IL-17A (clone eBio17B7; catalog no. 17-7177-81; Thermo Fisher Scientific). Because the intracellular-staining panels did not include CD3, CD3 gating was not applied to the FOXP3 or IL-17A analyses.

Separate single-stained control tubes were prepared for CD3, CD4, CD8, FOXP3, and IL-17A and were used to establish fluorescence compensation. Fluorescence-minus-one controls were not included in the original experiment. Positive gates were applied consistently to all samples using the gating boundaries established during the original analysis from the observed fluorescence distributions. No viability dye was included in the staining panels; therefore, a viability gate was not applied, and dead cells could not be directly excluded. Events were restricted according to lymphocyte light-scatter characteristics and singlet discrimination.

Approximately 10,000 gated events were acquired per sample, and the data were analyzed using FlowJo software (version 10.10.0; FlowJo LLC, Ashland, OR, USA). No ex vivo stimulation or protein-transport inhibitor was used before intracellular IL-17A staining. Accordingly, the measured populations are described as CD4^+^FOXP3^+^ and CD4^+^IL-17A^+^ phenotypes rather than as functionally validated regulatory T cells or T helper 17 cells. Representative sequential gating strategies are presented in Supplementary Figures S1 and S2. Each pig constituted one biological replicate, and individual flow-cytometric events were not treated as independent experimental observations.

Accordingly, all findings concerning CD4^+^FOXP3^+^ and CD4^+^IL-17A^+^ cells were treated as exploratory phenotypic observations. No inference was made regarding definitive regulatory T-cell or T helper 17 cell identity, cell viability, suppressive function, cytokine-secretion capacity, or cellular differentiation.

### 2.8. Isolation and Reverse Transcription of RNA and q-PCR

Total RNA was extracted from the duodenal, jejunal, and ileal mucosa using RNAiso Plus (catalog no. 9108; Takara Bio Inc., Kusatsu, Shiga, Japan) according to the manufacturer’s instructions. RNA concentration and purity were evaluated using a NanoDrop ND-2000 spectrophotometer. Complementary DNA was synthesized using the PrimeScript RT Reagent Kit with gDNA Eraser (catalog no. RR047Q; Takara Bio Inc.). The genomic DNA-removal step was followed by reverse transcription for 15 min at 37 °C and enzyme inactivation for 5 s at 85 °C.

Quantitative PCR amplification was performed using TB Green Premix Ex Taq II (Tli RNaseH Plus; catalog no. RR420L; Takara Bio Inc.) on a QuantStudio 6 Real-Time PCR System. The amplification program consisted of an initial denaturation at 95 °C for 30 s, followed by 40 cycles of 95 °C for 5 s and 60 °C for 30 s. A melting-curve analysis was conducted after amplification, and a single melting peak was required to confirm amplification specificity.

Primer performance was evaluated using standard curves prepared from serial dilutions of representative complementary DNA. The amplification efficiencies of all primer pairs were within 90–110%. β-Actin cycle-threshold values were compared among the experimental groups and showed no treatment-associated variation; therefore, β-actin was used as the reference gene. Primer sequences and related information are presented in Table A1.

Each biological sample was analyzed in three technical PCR wells. For each pig, intestinal segment, and gene, the cycle-threshold values from the three technical wells were averaged separately for the target and reference genes. The mean cycle-threshold value of the target gene was then normalized to that of β-actin as follows: ΔCt = mean Cttarget—mean Ctβ-actin. The ΔΔCt value was calculated relative to the mean ΔCt of the control group, and relative transcript abundance was expressed as 2^−^^ΔΔCt^ [36]. The resulting 2^−ΔΔCt^ value for each pig, gene, and intestinal segment constituted one animal-level biological observation and was entered into the statistical analysis. Technical PCR wells were not treated as independent experimental units.

### 2.9. Sample Size Calculation and Statistical Analysis

The study comprised 32 pigs, initially providing eight independent experimental units for each of the four treatment combinations. This sample size was selected based on previous experience with comparable Diquat-challenged weaned-pig experiments conducted in the same laboratory. An a priori power calculation was not performed. The individual pig was considered the experimental unit and biological replicate throughout the study. The actual number of animals included in each analysis is reported in the corresponding table or figure legend.

Before statistical analysis, technical subsamples obtained from the same animal were reduced to one animal-level observation for each endpoint. Where applicable, replicate assay wells were averaged before calculation of the final animal-level value. Multiple microscopic fields or tissue sections obtained from the same pig were similarly averaged to obtain one value per pig for each histological or imaging endpoint. For RT-qPCR analysis, the mean cycle-threshold values from the three technical wells were used to calculate one 2^−ΔΔCt^ value for each pig, gene, and intestinal segment. For flow-cytometric endpoints, one gated cell-frequency value was obtained for each pig and lymphoid tissue. Technical assay wells, microscopic fields, tissue sections, and individual flow-cytometric events were not treated as independent experimental units.

IBM SPSS Statistics for Windows, version 24.0 (IBM Corp., Armonk, NY, USA), was used for all inferential analyses. Each outcome was analyzed using a two-way analysis-of-variance model corresponding to the 2 × 2 factorial design. Dietary EGCG supplementation, Diquat challenge, and their interaction were included as fixed effects. The individual pig was considered the experimental unit and contributed one animal-level observation to each model.

For outcomes measured once per animal or in a single tissue, one 2 × 2 factorial model was fitted for each outcome. For outcomes measured in the duodenum, jejunum, and ileum, the same 2 × 2 factorial model was fitted separately within each intestinal segment. Similarly, flow-cytometric outcomes obtained from the mesenteric lymph nodes and spleen were analyzed separately within each lymphoid tissue.

Intestinal segment and lymphoid tissue were not included as factors in these models, and no statistical comparisons among intestinal segments or between lymphoid tissues were performed. Consequently, the actual analyses did not include intestinal segment as a repeated-measures factor, pig as a random subject effect, a segment or tissue main effect, treatment-by-segment or treatment-by-tissue interactions, or a within-animal covariance structure. The previous description of a repeated-measures mixed-effects model was inaccurate and has been corrected to reflect the segment- and tissue-specific 2 × 2 factorial analyses that were actually performed.

The EGCG × Diquat interaction was evaluated before interpretation of the corresponding main effects. When the interaction was not significant, the main effects of EGCG and Diquat were interpreted directly. When the interaction was significant, prespecified simple-effect comparisons were performed using estimated marginal means. These comparisons evaluated the effect of EGCG separately in nonchallenged and Diquat-challenged pigs and the effect of Diquat separately in pigs receiving the basal and EGCG-supplemented diets. Sidak adjustment was applied within the family of simple-effect comparisons for each individual outcome and intestinal segment or tissue.

The Sidak procedure was not intended to control multiplicity across all outcomes examined in the study. Because the outcomes represented distinct prespecified biological endpoints, no global multiplicity adjustment was applied across the complete set of endpoints. The reported *p* values are therefore outcome-specific and should be interpreted as exploratory evidence together with the direction and magnitude of the estimated effects and their 95% confidence intervals. Accordingly, the findings are interpreted as observed associations rather than as confirmatory evidence of a general treatment effect or demonstration of a specific biological mechanism.

Model residuals were assessed using the Shapiro–Wilk test, and homogeneity of variance was evaluated using Levene’s test. Statistical significance was defined as *p* < 0.05, whereas 0.05 ≤ *p* < 0.10 was described as a statistical tendency. Results are presented as means and standard errors of the mean. The expanded results from the segment- and tissue-specific 2 × 2 models, including the EGCG, Diquat, and EGCG × Diquat effect tests, effect estimates, 95% confidence intervals, adjusted *p* values for the applicable simple-effect comparisons, and analyzed sample sizes, are provided in Supplementary File S3.

## 3. Results

### 3.1. Effects of Dietary EGCG Supplementation and Diquat Challenge on Serum Antioxidant Status, Immunoglobulin Concentrations, and Inflammatory Cytokines

Dietary EGCG supplementation increased serum SOD activity (*p* = 0.002) and IgA, IgG, and IgM concentrations (*p* < 0.001, *p* < 0.001, and *p* = 0.007, respectively), whereas Diquat challenge decreased SOD activity (*p* = 0.021) and IgA, IgG, and IgM concentrations (*p* < 0.001, *p* < 0.001, and *p* = 0.005, respectively; Table 2). EGCG supplementation also decreased serum IL-1β (*p* = 0.033), whereas Diquat increased TNF-α (*p* = 0.037) and tended to increase IL-17 (*p* = 0.052). EGCG tended to increase serum CAT activity (*p* = 0.074).

A significant EGCG × Diquat interaction was detected for serum T-AOC (*p* = 0.020). Sidak-adjusted comparisons showed that EGCG increased T-AOC in Diquat-challenged pigs (adjusted *p* = 0.001). Diquat tended to decrease T-AOC in pigs fed the basal diet (adjusted *p* = 0.097), whereas the EGCG effect in non-challenged pigs and the Diquat effect in EGCG-supplemented pigs were not significant (adjusted *p* = 0.717 and 0.661, respectively). No significant main effects or interactions were detected for serum MDA, GSH-Px, or IL-6 (*p* > 0.05). Detailed model-based estimates, 95% confidence intervals, and simple-effect analyses are provided in Supplementary File S3 (Tables S1 and S2).

### 3.2. Effects of Dietary EGCG Supplementation and Diquat Challenge on Intestinal ZO-1 Immunoreactivity

**Figure 1:**
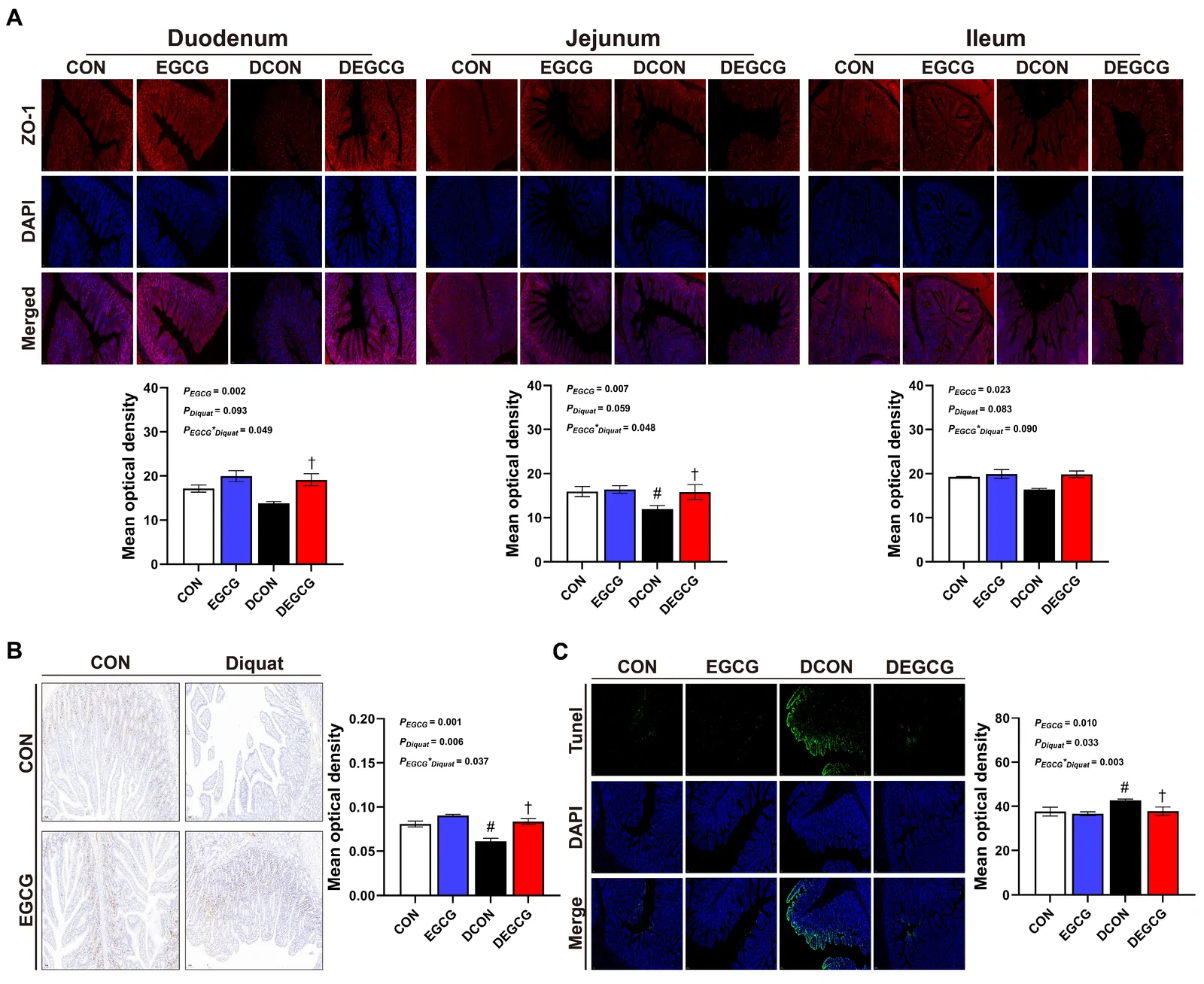
Effects of dietary EGCG supplementation and Diquat challenge on intestinal ZO-1 immunoreactivity, jejunal mucosal sIgA immunoreactivity, and jejunal epithelial TUNEL-positive signals in weaned pigs. (**A**) Representative immunofluorescence images and quantitative analysis of zonula occludens-1 (ZO-1) mean optical density in the duodenum, jejunum, and ileum. ZO-1 is shown in red, nuclei were counterstained with 4′,6-diamidino-2-phenylindole (DAPI) in blue, and merged images are presented. (**B**) Representative immunohistochemical images and quantitative analysis of secretory immunoglobulin A (sIgA) mean optical density in the jejunal mucosa. (**C**) Representative terminal deoxynucleotidyl transferase-mediated dUTP nick-end labeling (TUNEL) images and quantitative analysis of TUNEL-positive signal intensity in the jejunal epithelium. TUNEL-positive signals are shown in green, nuclei were counterstained with DAPI in blue, and merged images are presented. Images were acquired at 200× magnification. Data are presented as means ± SEM. For each pig, measurements from five non-overlapping microscopic fields in each of two non-consecutive sections were averaged to obtain one animal-level observation. The individual pig was considered the experimental unit. The *p* values presented above each outcome represent the model-based tests for the main effects of EGCG and Diquat and their interaction. For outcomes with a significant EGCG × Diquat interaction, symbols above the bars indicate the following simple-effect comparisons: * EGCG versus CON, ^†^ DEGCG versus DCON, and ^#^ DCON versus CON (Sidak-adjusted *p* < 0.05). Only significant comparisons are indicated. Exact adjusted *p* values, effect estimates, simultaneous 95% confidence intervals, and analyzed sample sizes are provided in Supplementary Tables S3 and S4. CON, pigs fed a basal diet; EGCG, pigs fed the basal diet supplemented with 50 mg/kg EGCG; DCON, pigs fed the basal diet and challenged with Diquat; DEGCG, pigs fed the EGCG-supplemented diet and challenged with Diquat; SEM, standard error of the mean.

Significant EGCG × Diquat interactions were detected for ZO-1 immunoreactivity in the duodenum and jejunum (*p* = 0.049 and 0.048, respectively; Figure 1A). EGCG increased ZO-1 immunoreactivity in Diquat-challenged pigs in both the duodenum and jejunum (adjusted *p* = 0.005 and 0.013, respectively). Diquat decreased jejunal ZO-1 immunoreactivity in pigs fed the basal diet (adjusted *p* = 0.034) and tended to decrease the corresponding duodenal value (adjusted *p* = 0.068). In the ileum, EGCG exerted a positive main effect on ZO-1 immunoreactivity (*p* = 0.023), whereas Diquat and the interaction showed decreasing trends (*p* = 0.083 and 0.090, respectively). Detailed model-based estimates, 95% confidence intervals, and simple-effect analyses are provided in Supplementary File S3 (Tables S3 and S4).

### 3.3. Effects of Dietary EGCG Supplementation and Diquat Challenge on Intestinal Mucosal sIgA Immunoreactivity

A significant interaction was detected for jejunal sIgA immunoreactivity (*p* = 0.037; Figure 1B). Diquat decreased sIgA immunoreactivity in pigs fed the basal diet (adjusted *p* = 0.006), whereas EGCG increased it in Diquat-challenged pigs (adjusted *p* = 0.003). The EGCG effect in non-challenged pigs was not significant (adjusted *p* = 0.276). Detailed model-based estimates, 95% confidence intervals, and simple-effect analyses are provided in Supplementary File S3 (Tables S3 and S4).

### 3.4. Effects of Dietary EGCG Supplementation and Diquat Challenge on Intestinal Epithelial TUNEL Staining

Jejunal TUNEL staining also showed a significant EGCG × Diquat interaction (*p* = 0.003; Figure 1C). Diquat increased TUNEL-positive staining in pigs fed the basal diet (adjusted *p* = 0.005), whereas EGCG reduced TUNEL-positive staining in Diquat-challenged pigs (adjusted *p* = 0.003). Neither the EGCG effect in non-challenged pigs nor the Diquat effect in EGCG-supplemented pigs was significant. Detailed model-based estimates, 95% confidence intervals, and simple-effect analyses are provided in Supplementary File S3 (Tables S3 and S4).

### 3.5. Effects of Dietary EGCG Supplementation on CD3^+^, CD4^+^, and CD8^+^ T-Cell Populations

**Figure 2:**
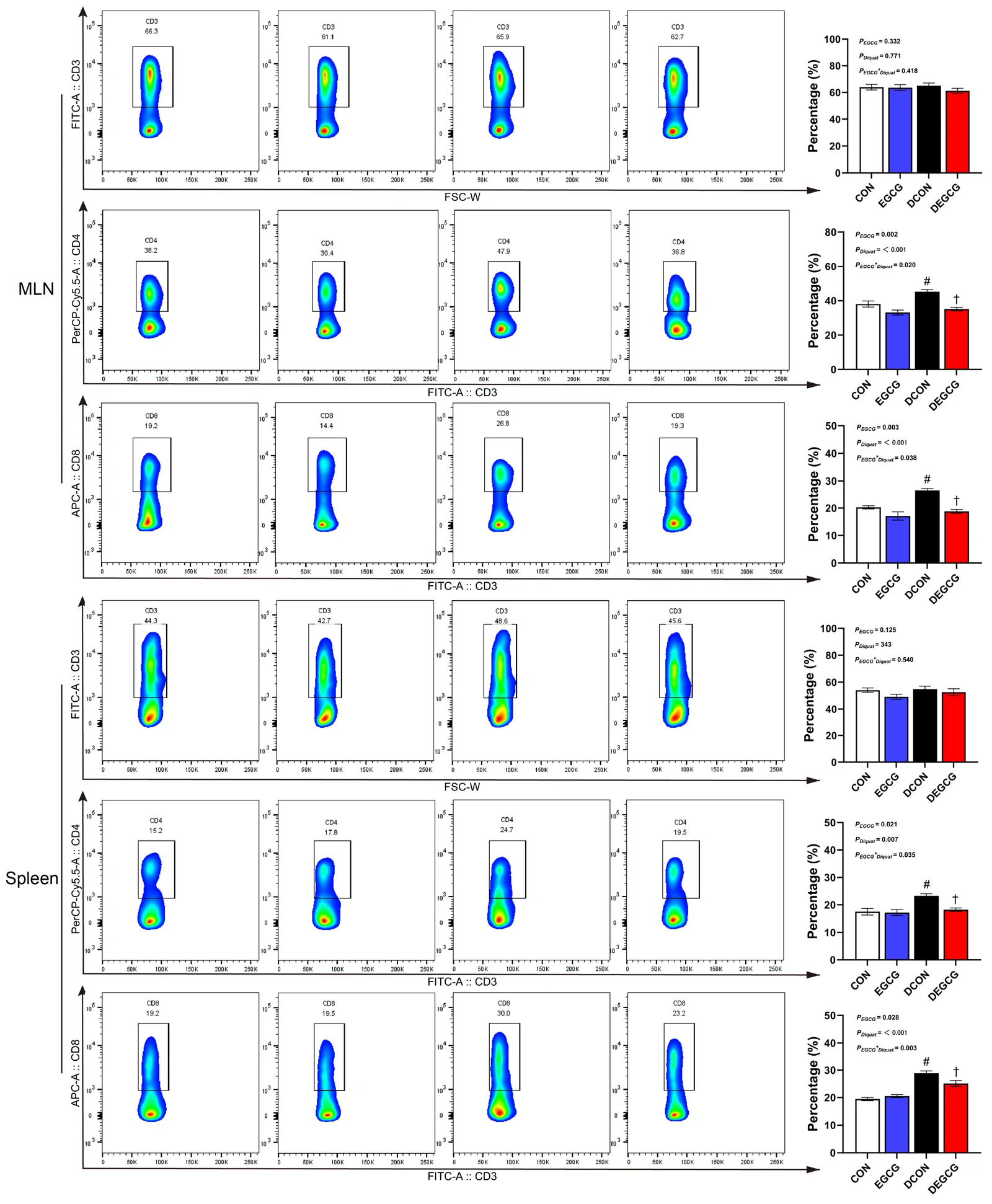
Effects of dietary EGCG supplementation and Diquat challenge on CD3^+^, CD4^+^, and CD8^+^ T-cell populations in the mesenteric lymph nodes and spleen of weaned pigs. Representative flow-cytometric plots and quantitative percentages of CD3^+^ T cells and CD4^+^ and CD8^+^ cells within the gated CD3^+^ T-cell population are presented for the mesenteric lymph nodes (MLN) and spleen. Data are presented as means ± SEM. Each pig constituted one biological replicate, and individual flow-cytometric events were not treated as independent observations. The *p* values presented above each outcome represent the model-based tests for the main effects of EGCG and Diquat and their interaction. For outcomes with a significant EGCG × Diquat interaction, symbols above the bars indicate the following simple-effect comparisons: * EGCG versus CON, ^†^ DEGCG versus DCON, and ^#^ DCON versus CON (Sidak-adjusted *p* < 0.05). Only significant comparisons are indicated. Exact adjusted *p* values, effect estimates, simultaneous 95% confidence intervals, and analyzed sample sizes are provided in Supplementary Tables S5 and S6. CON, pigs fed a basal diet; EGCG, pigs fed the basal diet supplemented with 50 mg/kg EGCG; DCON, pigs fed the basal diet and challenged with Diquat; DEGCG, pigs fed the EGCG-supplemented diet and challenged with Diquat; CD, cluster of differentiation; MLN, mesenteric lymph nodes; FSC-W, forward scatter width; FITC, fluorescein isothiocyanate; PerCP-Cy5.5, peridinin-chlorophyll-protein-Cyanine5.5; APC, allophycocyanin; SEM, standard error of the mean.

No significant EGCG, Diquat, or interaction effects were detected for the proportions of CD3^+^ cells in the mesenteric lymph nodes or spleen (*p* > 0.05; Figure 2).

Significant EGCG × Diquat interactions were detected for the proportions of CD4^+^ and CD8^+^ cells in the mesenteric lymph nodes (*p* = 0.020 and 0.038, respectively). Diquat increased both cell populations in pigs fed the basal diet (adjusted *p* = 0.003 and 0.006, respectively), whereas EGCG reduced their proportions in Diquat-challenged pigs (adjusted *p* = 0.002 for both).

In the spleen, interactions were also detected for CD4^+^ and CD8^+^ cells (*p* = 0.035 and 0.003, respectively). Diquat increased splenic CD4^+^ and CD8^+^ cell proportions in pigs fed the basal diet (adjusted *p* = 0.010 and *p* < 0.001, respectively), whereas EGCG decreased both populations in Diquat-challenged pigs (adjusted *p* = 0.020 and 0.005, respectively). Detailed model-based estimates, 95% confidence intervals, and simple-effect analyses are provided in Supplementary File S3 (Tables S5 and S6).

### 3.6. Exploratory Flow-Cytometric Observations of CD4^+^FOXP3^+^ and CD4^+^IL-17A^+^ Phenotypic Populations

**Figure 3:**
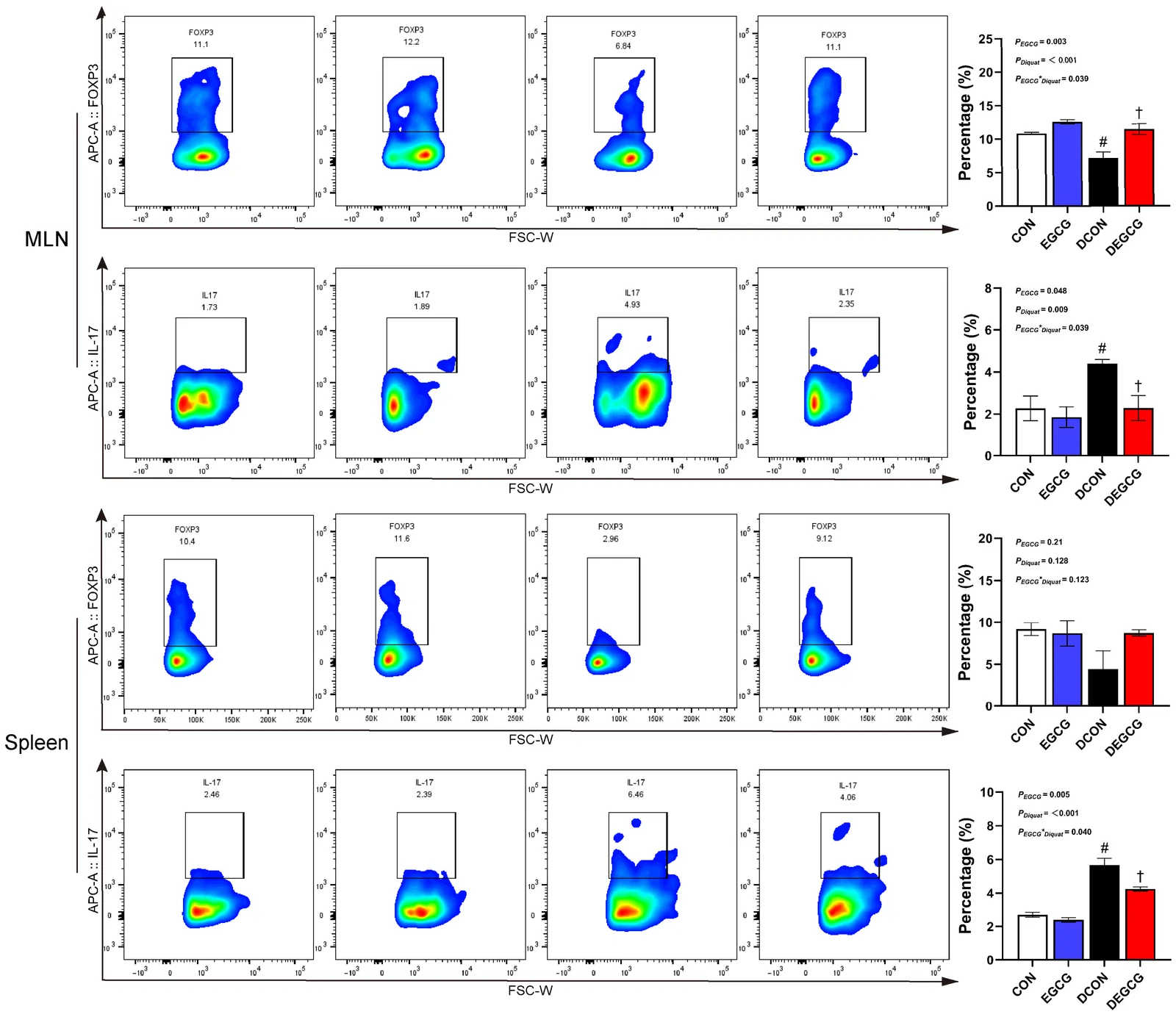
Effects of dietary EGCG supplementation and Diquat challenge on CD4^+^FOXP3^+^ and CD4^+^IL-17A^+^ cell populations in the mesenteric lymph nodes and spleen of weaned pigs. Representative flow-cytometric plots and quantitative percentages of CD4^+^FOXP3^+^ and CD4^+^IL-17A^+^ cells within the gated CD4^+^ cell population are presented for the mesenteric lymph nodes (MLN) and spleen. Data are presented as means ± SEM. Each pig constituted one biological replicate, and individual flow-cytometric events were not treated as independent observations. Because the intracellular-staining panels did not include CD3, viability staining, fluorescence-minus-one controls, ex vivo stimulation, protein-transport inhibition, or functional assays, these populations are presented as exploratory, marker-defined CD4^+^FOXP3^+^ and CD4^+^IL-17A^+^ phenotypes rather than as functionally validated regulatory T cells or T helper 17 cells. The *p* values presented above each outcome represent the model-based tests for the main effects of EGCG and Diquat and their interaction. For outcomes with a significant EGCG × Diquat interaction, symbols above the bars indicate the following simple-effect comparisons: * EGCG versus CON, ^†^ DEGCG versus DCON, and ^#^ DCON versus CON (Sidak-adjusted *p* < 0.05). Only significant comparisons are indicated. Exact adjusted *p* values, effect estimates, simultaneous 95% confidence intervals, and analyzed sample sizes are provided in Supplementary Tables S5 and S6. CON, pigs fed a basal diet; EGCG, pigs fed the basal diet supplemented with 50 mg/kg EGCG; DCON, pigs fed the basal diet and challenged with Diquat; DEGCG, pigs fed the EGCG-supplemented diet and challenged with Diquat; CD, cluster of differentiation; FOXP3, forkhead box P3; IL-17A, interleukin-17A; MLN, mesenteric lymph nodes; FSC-W, forward scatter width; APC, allophycocyanin; SEM, standard error of the mean.

In the exploratory flow-cytometric analysis, significant EGCG × Diquat interactions were detected for the frequencies of phenotypically defined CD4^+^FOXP3^+^ and CD4^+^IL-17A^+^ cells in the mesenteric lymph nodes (*p* = 0.039 for both; Figure 3). Diquat decreased the frequency of CD4^+^FOXP3^+^ cells and increased the frequency of CD4^+^IL-17A^+^ cells in pigs receiving the basal diet (adjusted *p* = 0.006 and 0.012, respectively). Conversely, EGCG increased the frequency of CD4^+^FOXP3^+^ cells and decreased that of CD4^+^IL-17A^+^ cells in Diquat-challenged pigs (adjusted *p* = 0.002 and 0.038, respectively).

No significant treatment effects were detected for splenic CD4^+^FOXP3^+^ cell frequency (*p* > 0.05). A significant EGCG × Diquat interaction was detected for splenic CD4^+^IL-17A^+^ cell frequency (*p* = 0.040). Diquat increased this phenotypic population in pigs receiving the basal diet (adjusted *p* < 0.001), whereas EGCG decreased it in Diquat-challenged pigs (adjusted *p* = 0.009). These results describe differences in marker-defined cell frequencies only and do not establish changes in functionally validated regulatory T cells or T helper 17 cells. Detailed model-based estimates, 95% confidence intervals, and simple-effect comparisons are provided in Supplementary Tables S5 and S6.

### 3.7. Effects of Dietary EGCG Supplementation and Diquat Challenge on Immune- and Apoptosis-Related Gene Expression in the Intestinal Mucosa

**Figure 4:**
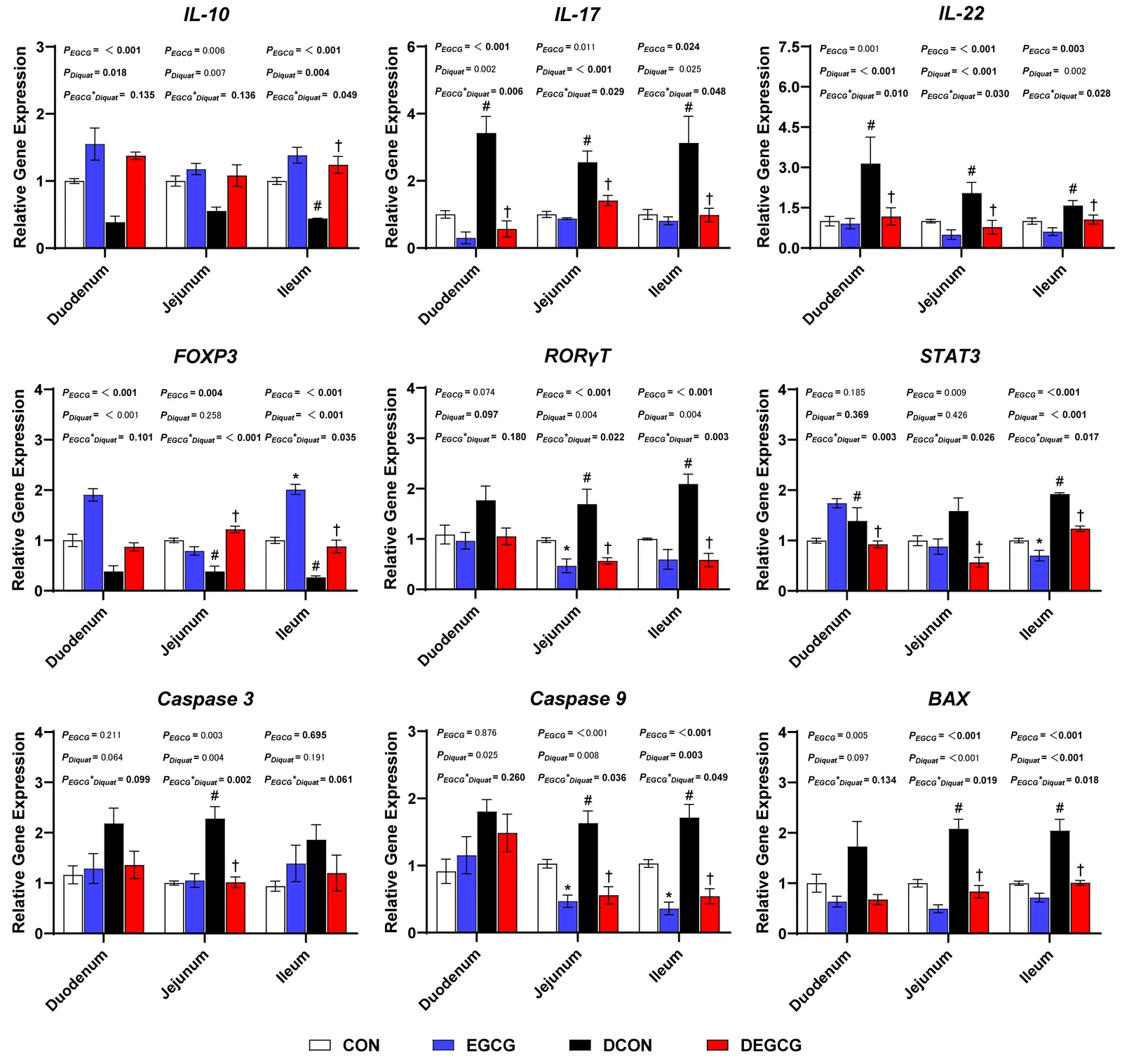
Effects of dietary EGCG supplementation and Diquat challenge on immune- and apoptosis-related gene expression in the intestinal mucosa of weaned pigs. The relative mRNA abundance of *IL-10*, *IL-17*, *IL-22*, *FOXP3*, *RORγT*, *STAT3*, *caspase-3*, *caspase-9*, and *BAX* was determined in the duodenal, jejunal, and ileal mucosa. Relative mRNA abundance was normalized to β-actin and calculated using the 2^−ΔΔCt^ method. Data are presented as means ± SEM. Technical PCR replicates were averaged to obtain one animal-level value for each pig, gene, and intestinal segment. The individual pig was considered the experimental unit. The *p* values presented above each intestinal segment represent the model-based tests for the main effects of EGCG and Diquat and their interaction. For gene × intestinal-segment outcomes with a significant EGCG × Diquat interaction, symbols above the bars indicate the following simple-effect comparisons: * EGCG versus CON, ^†^ DEGCG versus DCON, and ^#^ DCON versus CON (Sidak-adjusted *p* < 0.05). Only significant comparisons are indicated. Exact adjusted *p* values, effect estimates and simultaneous 95% confidence intervals are provided in Supplementary Tables S7 and S8. CON, pigs fed a basal diet; EGCG, pigs fed the basal diet supplemented with 50 mg/kg EGCG; DCON, pigs fed the basal diet and challenged with Diquat; DEGCG, pigs fed the EGCG-supplemented diet and challenged with Diquat; *IL-10*, interleukin-10; *IL-17*, interleukin-17; *IL-22*, interleukin-22; *FOXP3*, forkhead box P3; *RORγT*, retinoic acid receptor-related orphan receptor γt; *STAT3*, signal transducer and activator of transcription 3; *BAX*, BCL-2-associated X protein; SEM, standard error of the mean.

Intestinal immune-related gene-expression responses varied among intestinal segments (Figure 4; Supplementary Tables S7 and S8). In the duodenum and jejunum, EGCG increased *IL-10* expression (*p* < 0.001 and *p* = 0.006, respectively), whereas Diquat decreased *IL-10* expression (*p* = 0.018 and *p* = 0.007, respectively); the corresponding interactions were not significant (*p* = 0.135 and *p* = 0.136). In the ileum, an EGCG × Diquat interaction was detected for *IL-10* expression (*p* = 0.049). Diquat decreased ileal *IL-10* expression in pigs receiving the basal diet (Sidak-adjusted *p* = 0.009), whereas EGCG increased *IL-10* expression in Diquat-challenged pigs (adjusted *p* < 0.001).

Significant EGCG × Diquat interactions were detected for *IL-17* expression in the duodenum, jejunum, and ileum (*p* = 0.006, *p* = 0.029, and *p* = 0.048, respectively). In all three segments, Diquat increased *IL-17* expression in pigs receiving the basal diet (adjusted *p* = 0.002, *p* = 0.002, and *p* = 0.028, respectively), whereas EGCG decreased *IL-17* expression in Diquat-challenged pigs (adjusted *p* < 0.001, *p* = 0.012, and *p* = 0.027, respectively). Interactions were also detected for *IL-22* expression in the duodenum, jejunum, and ileum (*p* = 0.010, *p* = 0.030, and *p* = 0.028, respectively). Diquat increased *IL-22* expression in basal-diet pigs in all three segments (adjusted *p* < 0.001, *p* < 0.001, and *p* = 0.005, respectively), whereas EGCG decreased *IL-22* expression in Diquat-challenged pigs (adjusted *p* = 0.002, *p* = 0.002, and *p* = 0.004, respectively). In the duodenum, Diquat also increased *IL-22* expression in EGCG-supplemented pigs (adjusted *p* = 0.047). The remaining adjusted simple-effect comparisons for *IL-17* and *IL-22* were not significant.

In the duodenum, EGCG increased *FOXP3* expression and Diquat decreased it (both *p* < 0.001), without a significant interaction (*p* = 0.101). Significant interactions were detected in the jejunum and ileum (*p* < 0.001 and *p* = 0.035, respectively). In the jejunum, Diquat decreased *FOXP3* expression in basal-diet pigs (adjusted *p* = 0.002), whereas EGCG increased *FOXP3* expression in Diquat-challenged pigs (adjusted *p* < 0.001); Diquat also increased *FOXP3* expression within the EGCG-supplemented group (adjusted *p* = 0.019). In the ileum, EGCG increased *FOXP3* expression in both non-challenged and Diquat-challenged pigs (adjusted *p* < 0.001 and *p* = 0.001), whereas Diquat decreased *FOXP3* expression under both dietary conditions (both adjusted *p* < 0.001).

No significant main or interaction effects on duodenal *RORγT* expression were detected (all *p* ≥ 0.074). Significant interactions were observed in the jejunum and ileum (*p* = 0.022 and *p* = 0.003). Diquat increased *RORγT* expression in basal-diet pigs (adjusted *p* = 0.004 and *p* = 0.001), whereas EGCG decreased it in Diquat-challenged pigs in both segments (both adjusted *p* < 0.001). EGCG also decreased jejunal *RORγT* expression in non-challenged pigs (adjusted *p* = 0.024).

*STAT3* expression showed significant interactions in the duodenum, jejunum, and ileum (*p* = 0.003, *p* = 0.026, and *p* = 0.017, respectively). In the duodenum, EGCG increased *STAT3* expression in non-challenged pigs (adjusted *p* = 0.029), whereas Diquat decreased it in EGCG-supplemented pigs (adjusted *p* = 0.017). In the jejunum, EGCG decreased *STAT3* expression in Diquat-challenged pigs (adjusted *p* = 0.009), while the other simple-effect comparisons were not significant. In the ileum, EGCG decreased *STAT3* expression in both non-challenged and challenged pigs (adjusted *p* = 0.045 and *p* < 0.001), whereas Diquat increased it under both dietary conditions (adjusted *p* < 0.001 and *p* = 0.002).

For *caspase-3*, a significant interaction was detected in the jejunum (*p* = 0.002). Diquat increased jejunal *caspase-3* expression in basal-diet pigs (adjusted *p* = 0.001), whereas EGCG decreased it in Diquat-challenged pigs (adjusted *p* = 0.001). No significant main or interaction effects on *caspase-3* expression were detected in the duodenum or ileum (all *p* > 0.05). Diquat increased duodenal *caspase-9* expression as a main effect (*p* = 0.025). Significant interactions for *caspase-9* were detected in the jejunum and ileum (*p* = 0.036 and *p* = 0.049). In both segments, Diquat increased *caspase-9* expression in basal-diet pigs (adjusted *p* = 0.011 and *p* = 0.004), whereas EGCG decreased it in both non-challenged pigs (both adjusted *p* = 0.005) and Diquat-challenged pigs (both adjusted *p* < 0.001). EGCG decreased duodenal *BAX* expression as a main effect (*p* = 0.005), without a significant Diquat effect or interaction. Significant interactions for *BAX* were detected in the jejunum and ileum (*p* = 0.019 and *p* = 0.018). Diquat increased *BAX* expression in basal-diet pigs (adjusted *p* = 0.001 and *p* = 0.002), whereas EGCG decreased it in Diquat-challenged pigs (adjusted *p* < 0.001 and *p* = 0.002). The remaining adjusted simple-effect comparisons for *BAX* were not significant.

## 4. Discussion

The present study evaluated intestinal epithelial, mucosal immune, and apoptosis-related responses to dietary EGCG supplementation in Diquat-challenged weaned pigs. The principal findings were treatment-related alterations in ZO-1 and sIgA immunoreactivity, intestinal epithelial apoptosis, the frequencies of CD4^+^IL-17A^+^ and CD4^+^FOXP3^+^ cells, and the expression of genes associated with inflammatory signaling, CD4^+^ T-cell differentiation, and apoptosis. These findings provide a tissue-level characterization of the intestinal response to EGCG supplementation and Diquat challenge. Growth-performance outcomes from the same animal cohort have been reported previously [34] and are therefore not reconsidered here.

The serum results indicated that Diquat challenge disturbed systemic antioxidant and immune status, whereas dietary EGCG supplementation was associated with improved antioxidant defense and immunoglobulin concentrations. The significant EGCG × Diquat interaction for serum total antioxidant capacity indicated that the effect of EGCG depended on the challenge condition. Specifically, EGCG supplementation increased total antioxidant capacity in Diquat-challenged pigs, whereas the reduction induced by Diquat in pigs fed the basal diet represented a statistical tendency rather than a significant simple effect. In addition, the positive main effect of EGCG and the negative main effect of Diquat on serum superoxide dismutase activity suggested that EGCG was generally associated with enhanced enzymatic antioxidant defense. These effects may be related to the radical-scavenging properties of EGCG and its potential to support endogenous antioxidant systems; however, the present measurements do not establish a specific molecular mechanism. Dietary EGCG supplementation increased serum immunoglobulin A, immunoglobulin G, and immunoglobulin M concentrations, whereas Diquat challenge decreased these indices. Because no significant EGCG × Diquat interactions were detected for the immunoglobulins, these findings represent independent main effects rather than a challenge-specific restoration by EGCG. The cytokine responses were also selective. Dietary EGCG decreased serum interleukin-1β concentration, whereas Diquat increased tumor necrosis factor-α concentration and tended to increase interleukin-17 concentration. No significant effects were detected for serum interleukin-6, and no EGCG × Diquat interactions were observed for the measured cytokines. Therefore, the present results do not support a generalized systemic anti-inflammatory effect of EGCG but suggest that its association with circulating inflammatory mediators differed among individual cytokines. Previous studies investigating other dietary flavonoids provide a broader context for these findings. In lipopolysaccharide-challenged growing pigs, dietary quercetin supplementation increased selected immune indices, including immunoglobulin G concentrations and lymphocyte proportions, and reduced circulating interleukin-6 concentrations [37]. In broilers, graded quercetin supplementation primarily improved growth efficiency and nutrient utilization [38]. Although the latter study was conducted in poultry, these findings indicate that the biological effects of dietary flavonoids may depend on the animal species, compound structure, dietary dose, physiological status, and experimental challenge. Recent reviews have similarly concluded that phytogenic feed additives may support antioxidant defense, immune function, and intestinal health in non-ruminant animals [39] and pigs [40], although their efficacy varies considerably among studies and their mechanisms require further validation. In the present study, the improvements in serum antioxidant activity and immunoglobulin concentrations, together with the selective cytokine responses, further support a context-dependent rather than universal systemic effect of dietary EGCG. Differences in flavonoid structure, dietary dose, challenge type, and sampling time may partly explain the variation between the present findings and previous studies.

The intestinal barrier depends on both tight junction-associated epithelial integrity and effective mucosal immune defense [41]. In the present study, significant EGCG × Diquat interactions for duodenal and jejunal ZO-1 immunoreactivity showed that EGCG supplementation increased ZO-1 immunoreactivity in Diquat-challenged pigs. Diquat significantly reduced jejunal ZO-1 immunoreactivity in pigs fed the basal diet, whereas its corresponding effect in the duodenum was a statistical tendency. In the ileum, EGCG exerted a significant positive main effect on ZO-1 immunoreactivity, whereas the Diquat main effect and EGCG × Diquat interaction showed statistical tendencies. These observations indicate that the barrier-related responses varied among intestinal regions. ZO-1 is a scaffolding protein that coordinates tight-junction assembly and epithelial morphogenesis [42], whereas sIgA constitutes an important component of intestinal mucosal defense [43]. The significant interaction observed for sIgA further showed that Diquat reduced mucosal sIgA immunoreactivity in pigs fed the basal diet, whereas EGCG supplementation increased sIgA immunoreactivity specifically in Diquat-challenged pigs. The corresponding EGCG effect in non-challenged pigs was not significant. These findings suggest that EGCG supplementation may support both epithelial and mucosal immune components of the intestinal barrier under oxidative stress. This interpretation is broadly consistent with studies showing that plant polyphenols are associated with improved intestinal barrier-related outcomes in weaned pigs [41,44,45].

Intestinal epithelial apoptosis is an important feature of oxidative stress-induced mucosal injury because excessive epithelial cell loss can disrupt epithelial continuity and accelerate barrier dysfunction [46]. In the present study, the significant EGCG × Diquat interaction for TUNEL-positive signals showed that Diquat increased apoptotic signals in pigs fed the basal diet, whereas EGCG supplementation attenuated this increase in challenged pigs. Oxidative stress has been reported to promote epithelial cell death through ROS accumulation, mitochondrial dysfunction, and activation of apoptosis-related processes [47]. Previous studies have also associated plant-derived polyphenols with reduced intestinal epithelial apoptosis under oxidative stress conditions [48,49]. Accordingly, the present TUNEL results support an association between EGCG supplementation and reduced epithelial apoptotic injury. However, because TUNEL staining does not identify the specific molecular pathway responsible for cell death, the underlying anti-apoptotic mechanism cannot be established from these findings alone.

The changes in immune-cell populations provide evidence that dietary EGCG supplementation helped preserve immune homeostasis in the MLN and spleen of Diquat-challenged pigs. Although the percentage of total CD3^+^ T cells remained stable, significant EGCG × Diquat interactions were detected for both CD4^+^ and CD8^+^ T-cell populations in the two immune tissues. Diquat challenge increased these populations in pigs fed the basal diet, whereas EGCG supplementation reduced their frequencies in challenged pigs. However, Diquat continued to exert a significant effect on the splenic CD8^+^ population in EGCG-supplemented pigs. Thus, the results indicate partial and tissue-dependent modulation rather than complete normalization of all lymphocyte populations. This finding is consistent with previous evidence that dietary tea polyphenols modulate cell-mediated immune responses in Diquat-challenged piglets [50].

ROS are closely involved in CD4^+^ T-cell differentiation and polarization [51,52], and previous studies have associated EGCG with Treg- and Th17-related responses in inflammatory models [53,54,55]. In the present exploratory flow-cytometric analysis, Diquat decreased the frequency of phenotypically defined CD4^+^FOXP3^+^ cells and increased that of CD4^+^IL-17A^+^ cells in the mesenteric lymph nodes of pigs receiving the basal diet. EGCG was associated with changes in the opposite direction in Diquat-challenged pigs. In the spleen, no significant treatment effects were detected for CD4^+^FOXP3^+^ cell frequency, whereas EGCG was associated with a lower frequency of CD4^+^IL-17A^+^ cells under Diquat challenge. These findings represent tissue-dependent differences in marker-defined cell frequencies and should not be interpreted as evidence that EGCG altered the differentiation or function of regulatory T cells or T helper 17 cells. Because viability staining and fluorescence-minus-one controls were not included, the possible contribution of non-viable cells and uncertainty in positive-gate placement cannot be fully excluded. Furthermore, the absence of ex vivo stimulation, protein-transport inhibition, and functional validation precluded assessment of cytokine-secretion capacity, suppressive activity, or definitive T-cell identity. The CD4^+^FOXP3^+^ and CD4^+^IL-17A^+^ findings should therefore be regarded solely as exploratory phenotypic observations.

The intestinal mucosal gene-expression results further showed that dietary EGCG supplementation was associated with segment-dependent changes in the transcriptional profiles of genes related to inflammatory responses, immune-cell differentiation, and apoptosis under oxidative stress conditions. Previous research in weaned pigs has similarly shown that dietary polyphenols can alleviate intestinal inflammatory responses and contribute to the maintenance of mucosal immune homeostasis [56]. For *IL-10*, EGCG increased expression and Diquat decreased expression in the duodenum and jejunum. In the ileum, the significant interaction was attributable primarily to reduced *IL-10* expression following Diquat challenge in pigs fed the basal diet and increased expression following EGCG supplementation in challenged pigs; the EGCG effect in non-challenged pigs represented only a statistical tendency.

*IL-17* and *IL-22* are key effector cytokines associated with Th17-related immune responses [57]. Significant EGCG × Diquat interactions were detected for *IL-17* and *IL-22* expression in all three intestinal segments. For both genes, Diquat increased expression in pigs receiving the basal diet, whereas EGCG decreased expression in Diquat-challenged pigs. In the duodenum, Diquat also increased *IL-22* expression in EGCG-supplemented pigs, suggesting that EGCG attenuated but did not completely prevent the Diquat-associated *IL-22* response at this site. Overall, these segment-specific transcriptional associations are consistent with modulation of Th17-related signalling; however, they do not establish a direct mechanism or a cell-specific effect.

Consistent with these cytokine-related changes, the expression of *RORγT*, *STAT3*, and *FOXP3* also varied according to intestinal segment and treatment condition. In the duodenum, the main effects of EGCG and Diquat on *RORγT* expression represented statistical tendencies, whereas stronger challenge-dependent effects were detected in the jejunum and ileum. In these latter segments, Diquat increased *RORγT* expression in pigs fed the basal diet, and EGCG reduced it in challenged pigs. The response of *STAT3* was more segment dependent. In the duodenum, EGCG increased *STAT3* expression in non-challenged pigs, whereas Diquat decreased it in EGCG-supplemented pigs. In the jejunum, EGCG decreased *STAT3* expression in Diquat-challenged pigs, while the other simple-effect comparisons were not significant. In the ileum, EGCG decreased *STAT3* expression in both non-challenged and Diquat-challenged pigs, whereas Diquat increased it within both dietary groups. The *FOXP3* response was similarly segment dependent: EGCG exerted a positive main effect and Diquat a negative main effect in the duodenum, whereas the jejunal and ileal responses were characterized by significant interactions. In the jejunum, Diquat decreased *FOXP3* expression in basal-diet pigs, whereas EGCG increased it in Diquat-challenged pigs; within EGCG-supplemented pigs, Diquat also increased *FOXP3* expression. In the ileum, EGCG increased *FOXP3* expression in both non-challenged and Diquat-challenged pigs, whereas Diquat decreased it within both dietary groups. *RORγT* is a principal transcriptional regulator of Th17 differentiation, while *STAT3* promotes *RORγT* expression and *IL-17* production [58]. In contrast, *FOXP3* is a key transcription factor associated with regulatory T-cell development and can oppose Th17-related transcriptional activity [59]. Taken together, the observed expression patterns were generally compatible with lower pro-inflammatory and greater regulatory-associated transcriptional features following EGCG supplementation, particularly under Diquat challenge. However, the segment-specific responses of *FOXP3* and *STAT3* indicate that this interpretation does not apply uniformly to every gene or intestinal region. These transcriptional changes were broadly consistent with the flow-cytometric observations in the MLN and spleen. Nevertheless, because these outcomes were assessed at the mRNA level, they should be interpreted as changes in immune-related transcriptional profiles rather than direct evidence of altered T-cell differentiation or pathway activity.

The apoptosis-related transcriptional results partly complemented the changes in epithelial TUNEL-positive staining. Previous studies in Diquat-challenged weaned pigs demonstrated that dietary polyphenols reduced the expression of apoptosis-related genes in the small intestinal mucosa, supporting the importance of limiting epithelial apoptosis during oxidative stress [44]. The present responses were not uniform across the three intestinal segments. For *caspase-3*, a significant interaction was detected in the jejunum, where Diquat increased expression in pigs fed the basal diet and EGCG reduced expression in challenged pigs. No significant main or interaction effects on *caspase-3* expression were detected in the duodenum or ileum. For *caspase-9*, Diquat exerted a positive main effect in the duodenum, whereas significant interactions occurred in the jejunum and ileum. In both latter segments, Diquat increased *caspase-9* expression in pigs fed the basal diet, and EGCG decreased its expression in challenged pigs. EGCG also decreased *caspase-9* expression in non-challenged jejunal and ileal tissues.

For *BAX*, the duodenal results showed a negative main effect of EGCG without a significant interaction. In the jejunum and ileum, significant interactions indicated that Diquat increased *BAX* expression in pigs fed the basal diet, whereas EGCG reduced it in Diquat-challenged pigs. The reduction produced by EGCG in non-challenged pigs did not reach significance in either segment. *BAX*, *caspase-9*, and *caspase-3* are established components associated with mitochondrial apoptosis [60]. Therefore, the segment- and condition-specific reductions in their mRNA abundance, together with the lower TUNEL-positive signal in EGCG-supplemented challenged pigs, are consistent with reduced apoptosis-related responses. They do not, however, demonstrate uniform suppression of apoptosis-related gene expression throughout the intestine. Because activation of the corresponding proteins and signaling pathways was not directly measured, the present data do not establish suppression of the mitochondrial apoptotic pathway as a mechanism of EGCG action.

Several limitations of the present study should be acknowledged. The relatively small sample size, evaluation of only one dietary EGCG dose, use of an acute Diquat challenge model, and assessment primarily at a single terminal sampling time point may limit the precision, generalizability, and temporal interpretation of the findings. Several outcomes were evaluated using immunostaining, flow-cytometric cell frequencies, and mRNA abundance without corresponding protein-level, pathway-activation, or functional validation, while TUNEL staining was quantified as signal intensity rather than apoptotic-cell frequency. Although all eight pigs per treatment were included in the flow-cytometric analyses, no viability staining or fluorescence-minus-one controls were used, and the intracellular-staining panels did not include CD3. Moreover, CD4^+^IL-17A^+^ cells were assessed without ex vivo stimulation or a protein-transport inhibitor, and no functional assays were performed. Therefore, the CD4^+^FOXP3^+^ and CD4^+^IL-17A^+^ findings should be interpreted solely as exploratory, marker-defined phenotypic observations rather than as evidence of functionally validated regulatory T cells or T helper 17 cells. In addition, no global multiplicity adjustment was applied across the numerous outcomes examined; consequently, the results should be considered outcome-specific evidence and interpreted together with effect estimates and confidence intervals. Finally, the concurrent changes in antioxidant status, epithelial indices, immune-cell phenotypes, and gene expression do not establish causal relationships, and further dose–response, longitudinal, protein-level, viability-controlled flow-cytometric, and functional studies are required.

## 5. Conclusions

Dietary supplementation with 50 mg/kg EGCG was associated with less pronounced Diquat-related changes in systemic antioxidant and immune indices, intestinal epithelial barrier-related characteristics, and TUNEL-positive staining in weaned pigs. Exploratory flow-cytometric and transcriptional analyses also identified tissue- and intestinal-segment-dependent associations between EGCG supplementation and immune-cell phenotypes and mucosal gene-expression responses. Because numerous outcomes were examined without a global multiplicity adjustment, these findings should be considered outcome-specific exploratory evidence. The concurrent changes do not establish that EGCG acts through a specific antioxidant, immune, or apoptosis-related mechanism. Independent dose–response studies incorporating protein-level, pathway-level, and functional validation are required to confirm these observations and investigate the underlying mechanisms.

## Figures and Tables

**Table 1:** Experiment basal diet composition and nutrient level (as-fed basis).

Ingredients	%	Nutrient Level	Contents
Maize	28.31	Dry Matter, %	93.68
Extruded maize	24.87	Digestible energy, calculated, MJ/kg	14.78
Soybean meal	8.50	Crude Protein, %	19.68
Extruded full-fat soybean	10.30	Calcium, %	0.81
Fish meal	4.20	Available phosphorus, %	0.55
Whey powder	7.00	Lysine	1.35
Soybean protein concentrate	8.00	Methionine	0.42
Soybean oil	2.00	Methionine + cysteine	0.60
Sucrose	4.00	Threonine	0.79
Limestone	0.90	Tryptophan	0.22
Dicalcium phosphate	0.50		
NaCl	0.30		
L-Lysine HCl, 78%	0.47		
DL-Methionine	0.15		
L-Threonine, 98.5%	0.13		
Tryptophan, 98%	0.03		
Choline chloride	0.10		
Vitamin premix ^1^	0.04		
Mineral premix ^2^	0.20		
Total	100		

^1^ Vitamin premix provided the following per kg of diet: 9000 IU of VA, 3000 IU of VD_3_, 20 IU of VE, 3 mg of VK_3_, 1.5 mg of VB_1_, 4 mg of VB_2_, 3 mg of VB_6_, 0.02 mg of VB_12_, 30 mg of niacin, 15 mg of pantothenic acid, 0.75 mg of folic acid, and 0.1 mg of biotin. ^2^ The mineral premix provided the following per kg of diet: 100 mg Fe, 6 mg Cu, 100 mg Zn, 4 mg Mn, 0.30 mg I, 0.3 mg Se. The diet was formulated based on the recommendation of NRC2012 [35].

**Table 2:** Effects of dietary EGCG supplementation on serum antioxidant status, immunoglobulin levels, and inflammatory cytokines in Diquat-challenged weaned pigs.

Items	Treatments				SEM	*p*-Value		
	CON	EGCG	DCON	DEGCG		EGCG	Diquat	EGCG × Diquat
T-AOC (U/mL)	2.34	2.85	1.21	3.39 ^†^	0.25	0.001	0.371	0.020
CAT (U/mL)	6.56	8.27	3.82	7.81	0.77	0.074	0.286	0.439
MDA (nmol/mL)	2.09	2.46	2.66	2.01	0.18	0.711	0.874	0.203
T-SOD (U/mL)	63.03	75.24	38.78	70.06	4.38	0.002	0.021	0.112
GSH-Px (U/mL)	2799.07	2654.82	2608.30	2909.16	151.23	0.820	0.926	0.522
IgA (μg/mL)	1.86	2.13	1.56	1.89	0.06	<0.001	<0.001	0.556
IgG (μg/mL)	19.67	23.41	14.24	18.55	1.02	<0.001	<0.001	0.667
IgM (μg/mL)	2.20	2.45	1.79	2.18	0.08	0.007	0.005	0.462
IL-1β (pg/mL)	42.69	29.07	64.41	35.40	5.35	0.033	0.129	0.380
IL-6 (pg/mL)	103.17	101.08	121.93	99.90	4.33	0.167	0.300	0.244
IL-17 (pg/mL)	2.69	2.42	4.02	2.94	0.25	0.133	0.052	0.343
TNF-α (pg/mL)	10.51	12.12	28.32	19.98	3.06	0.532	0.037	0.362

Values are presented as means and pooled standard error of the mean (SEM), with eight pigs per treatment (*n* = 8). The individual pig was considered the experimental unit. The *p* values for EGCG, Diquat, and EGCG × Diquat represent the main effects of dietary EGCG supplementation, Diquat challenge, and their interaction, respectively. When the EGCG × Diquat interaction was significant (*p* < 0.05), selected simple-effect comparisons are indicated as follows: * EGCG versus CON, ^†^ DEGCG versus DCON, and ^#^ DCON versus CON (Sidak-adjusted *p* < 0.05). Only significant simple-effect comparisons are marked. Complete simple-effect estimates, simultaneous 95% confidence intervals, and exact Sidak-adjusted *p* values, including the DEGCG versus EGCG comparison, are provided in Supplementary Table S2. T-AOC, total antioxidant capacity; CAT, catalase; MDA, malondialdehyde; SOD, superoxide dismutase; GSH-Px, glutathione; IgA, immunoglobulin A; IgG, immunoglobulin G; IgM, immunoglobulin M; IL-1β, interleukin-1β; IL-6, interleukin-6; IL-17, interleukin-17; TNF-α, tumor necrosis factor-α; CON, pigs fed a basal diet; EGCG, pigs fed the basal diet supplemented with 50 mg/kg EGCG; DCON, pigs fed the basal diet and challenged with Diquat; DEGCG, pigs fed the EGCG-supplemented diet and challenged with Diquat.

## Data Availability

The original contributions presented in this study are included in the article/Supplementary Materials. Further inquiries can be directed to the corresponding authors.

## Supplementary Materials

The following supporting information can be downloaded at: https://www.mdpi.com/article/10.3390/antiox15101270/s1. Supplementary File S1: Figure S1, representative sequential gating strategies for CD3^+^, CD4^+^, and CD8^+^ cell populations in the mesenteric lymph nodes and spleen; Figure S2, representative sequential gating strategies for CD4^+^FOXP3^+^ and CD4^+^IL-17A^+^ phenotypes in the mesenteric lymph nodes and spleen. Supplementary File S2: HPLC compositional verification of the EGCG product used in the animal experiment. Supplementary File S3: expanded statistical results from the outcome-specific 2 × 2 factorial models, including the main effects of EGCG and Diquat, their interaction, effect estimates, simultaneous 95% confidence intervals, Sidak-adjusted simple-effect comparisons where applicable, and analyzed sample sizes (Tables S1–S8).

## Abbreviations

ADFI	Average daily feed intake
ADG	Average daily gain
BAX	BCL-2-associated X protein
CAT	catalase
CON	pigs were fed with a basal diet
CP	crude protein
Ct	cycle threshold
DAPI	4′,6-diamidino-2-phenylindole
DCON	pigs were fed with a basal diet and challenged by Diquat
DEGCG	pigs were fed with an EGCG-containing diet and challenged by Diquat
DM	dry matter
EGCG	epigallocatechin-3-gallate
EE	ether extract
FOXP3	forkhead box P3
FSC-A	forward scatter area
FSC-H	forward scatter height
GSH-Px	glutathione peroxidase
HPLC	high-performance liquid chromatography
IgA	immunoglobulin A
IgG	immunoglobulin G
IgM	immunoglobulin M
IL-1β	interleukin-1β
IL-6	interleukin-6
IL-10	interleukin-10
IL-17	interleukin-17
IL-22	interleukin-22
MDA	malondialdehyde
MLN	mesenteric lymph nodes
PBS	phosphate-buffered saline
RORγT	retinoic acid receptor-related orphan receptor γt
sIgA	secretory immunoglobulin A
SOD	superoxide dismutase
SSC-A	side scatter area
STAT3	signal transducer and activator of transcription 3
T-AOC	total antioxidant capacity
TNF-α	Tumor necrosis factor-α
TUNEL	terminal deoxynucleotidyl transferase-mediated dUTP nick-end labeling
ZO-1	zonula occludens-1

## Appendix A

**Table A1:** Sequences of primers for genes.

Gene	Primer Sequence (5′-3′)	Product Size (bp)	NCBI Reference Sequence
*β-Actin*	F: TGGAACGGTGAAGGTGACAGC	177	NM_001444420.1
	R: GCTTTTGGGAAGGCAGGGACT		
*IL-10*	F: TGCTCTATTGCCTGATCTTCCTG	157	NM_214041.1
	R: CCCATCTGGTCCTTCGTTTG		
*IL-17*	F: ACTCCAAACGCTTCACCTCACC	235	NM_001005729.1
	R: CAGCCCACTGTCACCATCACTT		
*IL22*	F: AGCTTGGAGAGAGTGGAGAGAT	75	XM_021091968.1
	R: TGCATTCATTCTTCAGGGCCATA		
*RORγT*	F: CATTCAGTACGTGGTGGAGTTCG	143	XM_013997125.2
	R: TGTGGTTGTCAGCGTTGTAGGC		
*Foxp3+*	F: TCTGACAAGGGTTCCTGCTGTATTG	219	NM_001128438.1
	R: GCGGATGAGGGTGGCGTATGT		
*STAT3*	F: TGGAGAAGGACATCAGCGGTAAG	142	NM_001044580.1
	R: ACCAGCGGAGACACAAGGATG		
*Caspase 3*	F: GGGATTGAGACGGACAGTGG	136	NM_214131.1
	R: TGAACCAGGATCCGTCCTTTG		
*Caspase 9*	F: AATGCCGATTTGGCTTACGT	195	XM_084962990.1
	R: CATTTGCTTGGCAGTCAGGTT		
*BAX*	F: AAGCGCATTGGAGATGAACT	121	XM_013998624.2
	R: TGCCGTCAGCAAACATTTC		

*IL-10*: interleukin-10, *IL-17*: interleukin-17, *IL-22*: interleukin-22, *FOXP3*: forkhead box P3, *RORγT*: retinoic acid receptor-related orphan receptor γt, *STAT3*: signal transducer and activator of transcription 3, *BCL-2*: B-cell lymphoma-2, *BAX*: Bcl-2-associated X protein.
