# Supplementary material for: Protective Effects of Dietary Epigallocatechin-3-Gallate in Weaned Pigs Exposed to Oxidative Stress
Source: Antioxidants (Basel). 2026 Oct 2;15(10):1270. doi: 10.3390/antiox15101270 (PMC13700602; doi:10.3390/antiox15101270)
Supplement: Supplementary file 1 [file antioxidants-15-01270-s001.zip › Supplementary File S1.pdf]

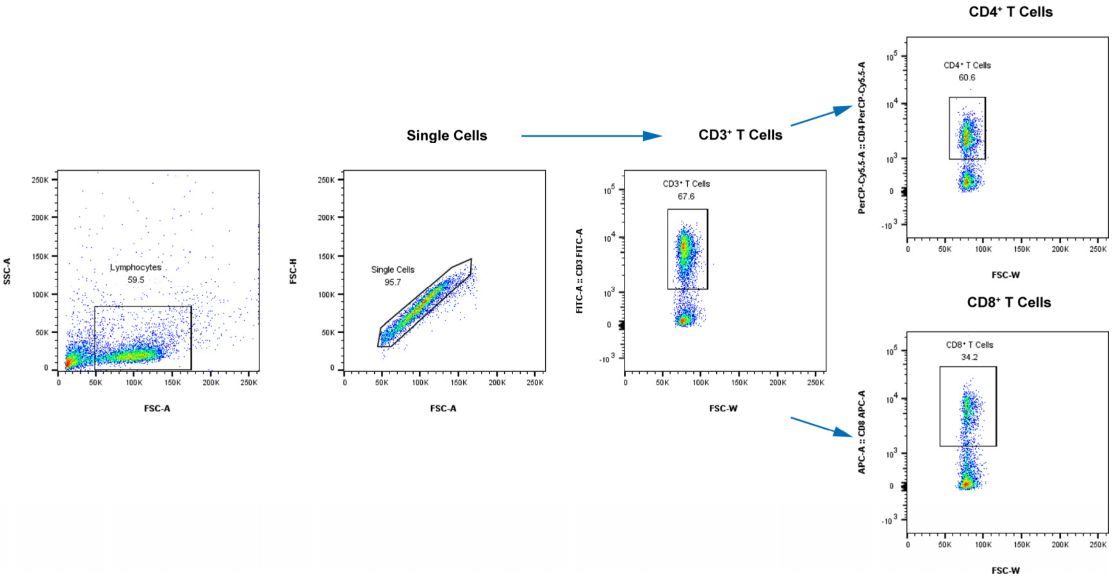

Figure S1. Representative gating strategy for CD3⁺, CD4⁺, and CD8⁺ T-cell populations in the mesenteric lymph nodes and spleen. Mesenteric lymph node and spleen samples were analyzed separately using the same gating strategy. Lymphocytes were first identified based on forward scatter area (FSC-A) and side scatter area (SSC-A), followed by singlet-cell gating using FSC-A and forward scatter height (FSC-H). CD3⁺ T cells were subsequently gated from the singlet lymphocyte population, and CD4⁺ and CD8⁺ T-cell populations were identified within the CD3⁺ T-cell gate. The plots shown are representative of the gating procedure applied to samples from the experimental pigs.

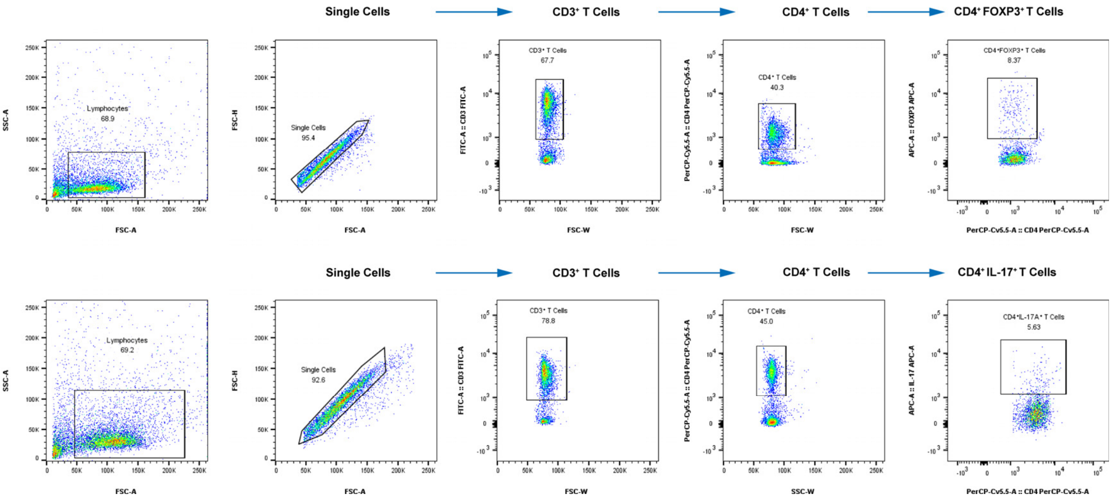

Figure S2. Representative flow-cytometric gating strategy for CD4⁺FOXP3⁺ and CD4⁺IL-17A⁺ phenotypes in the mesenteric lymph nodes and spleen of weaned pigs. Lymphocytes were initially identified according to forward-scatter area (FSC-A) and side-scatter area (SSC-A), followed by singlet discrimination using FSC-A versus forward-scatter height (FSC-H). CD4⁺ cells were subsequently gated, and FOXP3⁺ or IL-17A⁺ events were quantified separately within the gated CD4⁺ population after fixation and permeabilization. Representative fluorescence-minus-one controls used to define the FOXP3⁺ and IL-17A⁺ gates are presented. No viability dye was included in the staining panels; therefore, a viability gate

was not applied. The intracellular-staining panels did not include CD3, and no ex vivo stimulation or protein-transport inhibitor was used before IL-17A staining. Accordingly, the measured populations are described as $CD4^+FOXP3^+$ and $CD4^+IL\text{-}17A^+$ phenotypes rather than as functionally validated regulatory T-cell and T helper 17-cell populations. MLN, mesenteric lymph nodes; FOXP3, forkhead box P3; IL-17A, interleukin-17A; FMO, fluorescence minus one.
