# Supplementary material for: Protective Effects of Dietary Epigallocatechin-3-Gallate in Weaned Pigs Exposed to Oxidative Stress
Source: Antioxidants (Basel). 2026 Oct 2;15(10):1270. doi: 10.3390/antiox15101270 (PMC13700602; doi:10.3390/antiox15101270)
Supplement: Supplementary file 1 [file antioxidants-15-01270-s001.zip › Supplementary File S2.pdf]

HPLC Analysis Report for EGCG

English Translation of the Original Chinese Instrument Report

**Data file**	C0069360.D
**Sample name**	YP2026030086 PX
**Acquisition operator**	Li Yongfang
**Sample operator**	Li Yongfang
**Acquisition instrument**	Lyan-LC002
**Sequence line**	16
**Position**	38
**Injection date/time**	2026-03-05 04:06:16
**Number of injections**	1
**Injection volume**	5.000 uL
**Acquisition method**	EGCG (YD).M
**Acquisition method last modified**	2025-02-27 09:11:09
**Analysis method**	EGCG20260305-1.M
**Analysis method last modified**	2026-03-05 10:44:15

Chromatogram

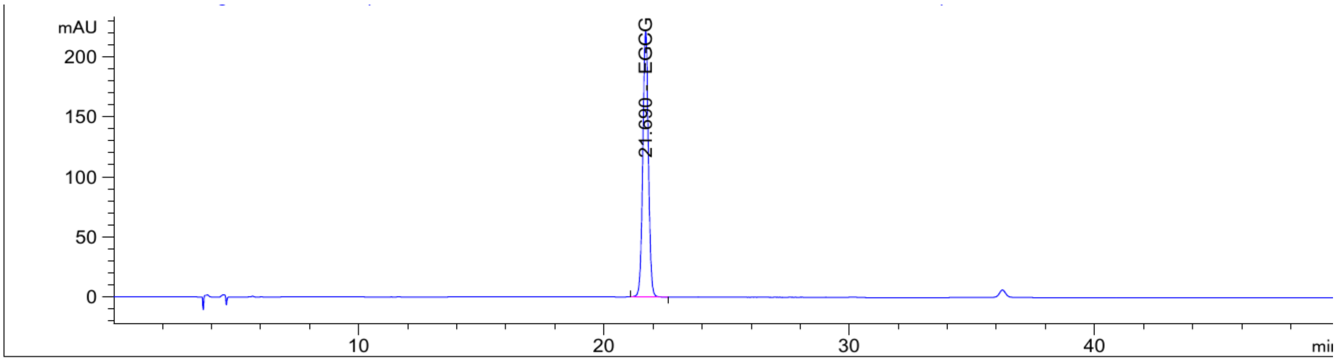

External Standard Percentage Report

**Sort**	Signal
**Calibration data modified**	2026-03-05 09:58:51
**Multiplier factor**	1.0000
**Dilution factor**	50.0000
**Sample amount**	22.68000 ng/uL
**Signal**	DAD1 A, Sig=280,4 Ref=off

Retention time (min)	Type	Peak area (mAU*s)	Amount/Peak area	Content (%)	Name
21.690	BB	3634.61084	1.21202e-4	95.174523	EGCG
				95.174523	Total

Reported EGCG content: **95.17%**

Retention time: 21.690 min; DAD detection at 280 nm; external-standard quantification.

Translation note

This English translation was prepared from the original Chinese HPLC instrument report. All numerical values are reproduced without alteration, and the chromatogram is reproduced from the original report. The original Chinese report remains the source document.
